# Compromised trigemino-coerulean coupling in migraine sensitization can be prevented by blocking beta-receptors in the locus coeruleus

**DOI:** 10.1186/s10194-023-01691-1

**Published:** 2023-12-08

**Authors:** Jérémy Signoret-Genest, Maxime Barnet, François Gabrielli, Youssef Aissouni, Alain Artola, Radhouane Dallel, Myriam Antri, Philip Tovote, Lénaïc Monconduit

**Affiliations:** 1 Université Clermont Auvergne, CHU Clermont-Ferrand, Inserm/UCA U1107, Neuro-Dol: Trigeminal Pain and Migraine, Faculté de Chirurgie Dentaire, 2 Rue de Braga, 63100 Clermont-Ferrand, France; 2 Institute of Clinical Neurobiology, University Hospital Würzburg, 97078 Würzburg, Germany; 3 Department of Psychiatry, Center of Mental Health, University Hospital Würzburg, 97078 Würzburg, Germany

**Keywords:** Medullary dorsal horn, Locus coeruleus, Adrenergic receptors, Migraine

## Abstract

**Background:**

Migraine is a disabling neurological disorder, characterized by recurrent headaches. During migraine attacks, individuals often experience sensory symptoms such as cutaneous allodynia which indicates the presence of central sensitization. This sensitization is prevented by oral administration of propranolol, a common first-line medication for migraine prophylaxis, that also normalized the activation of the locus coeruleus (LC), considered as the main origin of descending noradrenergic pain controls. We hypothesized that the basal modulation of trigeminal sensory processing by the locus coeruleus is shifted towards more facilitation in migraineurs and that prophylactic action of propranolol may be attributed to a direct action in LC through beta-adrenergic receptors.

**Methods:**

We used simultaneous in vivo extracellular recordings from the trigeminocervical complex (TCC) and LC of male Sprague–Dawley rats to characterize the relationship between these two areas following repeated meningeal inflammatory soup infusions. Von Frey Hairs and air-puff were used to test periorbital mechanical allodynia. RNAscope and patch-clamp recordings allowed us to examine the action mechanism of propranolol.

**Results:**

We found a strong synchronization between TCC and LC spontaneous activities, with a precession of the LC, suggesting the LC drives TCC excitability. Following repeated dural-evoked trigeminal activations, we observed a disruption in coupling of activity within LC and TCC. This suggested an involvement of the two regions’ interactions in the development of sensitization. Furthermore, we showed the co-expression of alpha-2A and beta-2 adrenergic receptors within LC neurons. Finally propranolol microinjections into the LC prevented trigeminal sensitization by desynchronizing and decreasing LC neuronal activity.

**Conclusions:**

Altogether these results suggest that trigemino-coerulean coupling plays a pivotal role in migraine progression, and that propranolol’s prophylactic effects involve, to some extent, the modulation of LC activity through beta-2 adrenergic receptors. This insight reveals new mechanistic aspects of LC control over sensory processing.

**Supplementary Information:**

The online version contains supplementary material available at 10.1186/s10194-023-01691-1.

## Background

Migraine, listed as the sixth most disabling disorder globally by the World Health Organization, and as the most disabling of all neurological disorders [1], is characterized by attacks of unilateral, throbbing head pain, with sensitivity to movement, visual, auditory, and other afferents inputs, such as cutaneous allodynia [2–4]. The presence and intensity of cephalic allodynia are linked to the frequency of migraine attacks [3] and are regarded as a possible contributing factor in the development of chronic migraines [5, 6], which is characterized by experiencing headache on more than 14 days per month for at least 3 months, with 8 of these exhibiting migraine features.

Cephalic allodynia reflects the sensitization of second-order trigeminovascular neurons receiving convergent inputs from the meninges and facial skin [7, 8]. Apart from peripheral inputs, the neuronal activity of the trigeminocervical complex (TCC) can be modulated by descending pain controls that originate from the brainstem, particularly from the locus coeruleus (LC) [9, 10]. The LC is known for its primary noradrenergic projections on the dorsal horn, which play a significant role in pain modulation [11]. An extensive bibliography exists on LC involvement in chronic pain (see [9] for review), but a global pattern is difficult to discern. The current theory is that the LC exerts an inhibitory control over sustained acute nociception, but that this control is lost with chronic pain.

On the other hand, systemic administration of the beta-blocker propranolol, one of the most commonly prescribed drugs for the prevention of migraine, reduces facial allodynia as well as associated TCC central sensitization [12], cortical spreading depression propagation [13, 14] and LC activation following repeated dural stimulations [12]. Propranolol inhibits NO production [15], but is ineffective in blocking neurogenic dural vasodilation [16] or plasma protein extravasation [17]. Being a highly lipophilic molecule [18], propranolol could reduce the frequency of migraine attacks through central effects. The role of beta adrenergic receptors (AR) in modulating of LC neurons activity has been relatively understudied [19, 20]. Indeed, most studies have focused on the involvement of alpha-2 AR, showing that LC neurons are under the influence of a tonically active inhibitory control mechanism mediated by these AR [21]. We hypothesized (i) that the LC, by facilitating TCC sensitization, promotes the progression of migraine and (ii) that propranolol could reduce TCC sensitization, by directly targeting the LC through beta AR. To test these hypotheses, we first characterized the LC-TCC relationship by performing double-site electrophysiological recordings in the TCC and the LC. We then examined whether such relationship was altered concomitantly with TCC central sensitization following repeated inflammatory soup (IS) infusions to dura mater, to recurrently activate and sensitize dural nociceptors. Next, we tested the effect of propranolol microinjections into the LC on central sensitization and on this LC-TCC relationship. Finally, to understand the mechanisms underlying the effects of propranolol in LC, we studied electrophysiological properties of LC neurons using whole-cell patch-clamp recordings and the expression of beta-1 and beta-2 AR into the LC using RNAscope in situ hybridization.

## Methods

### Animals

**Fig. 1:**
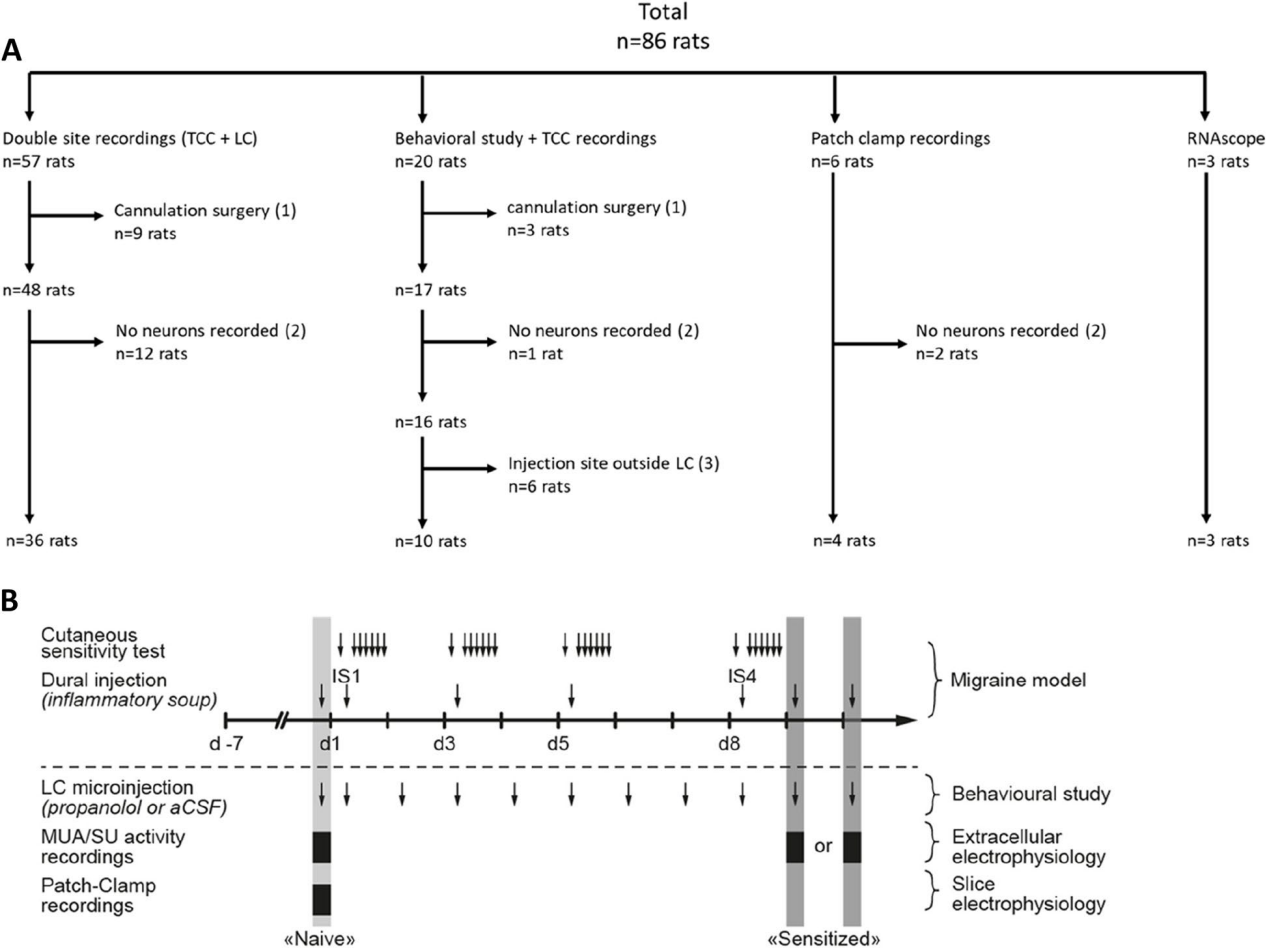
Flow chart of animal experiments and schematic representation of the experimental design. **A** Number of animals used in each experiment: (1) animals lost during the surgery of cannula placement or blocked cannula; (2) animals excluded during electrophysiological recordings because no neurons were recorded (animals lost during surgery, loss of signal, neurons not found); (3) animals excluded because the injection site of aCSF or propranolol were outside the LC. **B** Rats were acclimated to handling and sensory testing 7 days before and after the surgical implantation of a dural cannula (performed on D -7). In the migraine model group, rats received four injections of inflammatory soup (IS). Mechanical sensitivity of ophthalmic cutaneous territory was tested every day, one week after the surgery. For the combined behavioral/electrophysiological study, animals received, in addition to dural injection, a daily intra-LC microinjection of propranolol or aCSF. Electrophysiological recordings were performed either in naive (no surgery, no injections) or sensitized rats, in which case recordings were realised either one or two days after the fourth IS injection (IS4 in the rest of the manuscript)

Male Sprague–Dawley rats weighing 50 to 250 g (Charles River, L’Arbresle, France) were housed at 22.6 ± 1 °C in plastic cages (size: 425 × 266x185 mm; 2–3 rats per cage) on soft bedding with ad libitum water and food pellets under an inverted 12/12 h light/dark cycle for at least 1 week before the experiments. Every effort was made to minimize the number of animals used: numbers of animals were selected according to previous experience [7, 12], *i.e.* with a trade-off between reaching routine sample sizes for each type of experiments and minimizing numbers of animals for pain experiments. Experiments were performed on 86 animals (Fig. 1A). All experiments, analysis, and reporting were ARRIVE-compliant (Animals in research: reporting in vivo experiments). Animal experiments were performed according to the ethical guidelines set by the International Association for the Study of Pain [22] and European Directive 2010/63/EU on the protection of animals used for scientific purpose. The protocols applied here for animal care and use were approved by the Clermont Auvergne University Institutional Review Board and authorized by the French Ministry of Primary, Secondary and Higher Education, and Research (n ° CE 27–12, 26–12, 700.02).

### Experimental design

The study is divided in three parts. First, to investigate LC/TCC physiological relationship, double-site electrophysiology was performed in naive rats. Artificial cerebrospinal fluid (aCSF) was microinjected in the LC during recordings as a control condition for pharmacological manipulations; similar recordings were conducted in a rat model for the recurrent activation of dural nociceptors to assess potential changes. Second, to study the effects of propranolol on TCC sensitization, behavioral effects of propranolol microinjections in LC were assessed in the model. The same rats were subsequently subjected to electrophysiological recordings. Finally, to identify the preventive action of propranolol microinjection into the LC, RNAScope fluorescent in situ hybridization, in vivo double-site and whole-cell patch-clamp electrophysiological recordings were conducted in naive animals.

### Dural-evoked trigeminal activation

As previously described [7, 12], rats underwent surgery after one week of habituation to the experimenter and the observation room. After anaesthesia, a cannula was carefully inserted (1 mm anteroposterior and mediolateral from bregma) into a 0.5 mm diameter craniotomy, fixed to anchor screws with dental cement, and the skin was sutured. After surgery, rats were housed separately and recovered for at least one week before sensory testing while their state was closely monitored. Injections (10 μl) of IS or aCSF were performed under brief anaesthesia (3% isoflurane; ≤ 3min) through the intracranial cannula, four times, at 2 to 3-day intervals during the 8-day injection protocol (Fig. 1B).

### Intra-LC injections

During the surgery to implant the dural cannula, a second stainless-steel guide cannula (o.d. 0.6mm, i.d. 0.5mm) was implanted unilaterally in the brainstem, aimed at the LC ipsilateral to the first cannula (anteroposterior, –9.68 mm; mediolateral, 1.4 mm from bregma; dorsoventral, 6 mm), and fixed with the screws and dental acrylic used for the dural cannula. The post-surgery monitoring is the same as described above. Animals for the behavioral study received daily microinjections (250 nL) of aCSF or propranolol into the LC. The microinjections were performed under brief anesthesia (3% Isoflurane; ≤ 3min) using a needle (26 G) introduced through the guide cannula until its lower end extended 1mm below the guide cannula. The needle was left in place for one additional minute after the injection.

The days of concomitant administration of IS or aCSF on the dura, animals were anesthetized a single time, and microinjection was carried out 1 min before dural administration of aCSF or IS.

After each experiment, the location of the microinjection was confirmed by cryosectioning the brainstem. When the site was located outside the LC, the rat was excluded from the analysis.

### Behavioral testing

As described previously [7], the week before surgery, rats were acclimated to handling, to the behavioral context and to innocuous mechanical stimulation (sub-threshold to elicit the appropriate behavioral response). Mechanical cutaneous sensitivity of the face was then assessed before and after surgery to ensure the absence of surgery-induced sensitization, and every day during the 8-day injection protocol, on both IS injection and interictal days. Face static mechanical threshold were measured by applying von Frey (VF) hairs (Bioseb, France) to the midline of the forehead. For cephalic mechanical dynamic sensitivity, responses to gentle air-puff stimuli on the face were scored (dynamic mechanical scores: DMS) according to Vos et al*.* [23].

### In vivo extracellular recordings

After the animals were anesthetized in a box with 3% isoflurane in a N_2_O/O_2_ mixture (2:1), the trachea was cannulated and the carotid artery and external jugular vein catheterized. Animals were then paralyzed by an intravenous perfusion of vecuronium bromide and artificially ventilated with a volume-controlled pump. Levels of isoflurane, O_2_, N_2_O and end-tidal CO_2_ were measured by an anaesthetic gas analyser (Drager Vamos) during the entire experimental period. The arterial catheter was attached to a calibrated pressure transducer connected to an amplifier for continuous monitoring of the mean arterial blood pressure. The analog output from the blood pressure amplifier was connected to a computer data sampling system (Cambridge Electronic Design 1401plus interface, CED; Cambridge, UK). The animals were placed in a stereotaxic frame with the head fixed in a slightly ventroflexed position. A craniotomy was performed to allow chemical and electrical stimulation of the dura and passage of the electrode for LC recordings. After surgery, the level of isoflurane was progressively reduced to 1.2% in pure O_2_ and maintained at this level during the recording period.

Unitary extracellular recordings were made from the right TCC with glass micropipettes (0.5–2 MΩ) filled with a mixture of 5% NaCl and pontamine sky blue. TCC electrophysiology was conducted as previously described [7]. Briefly, wide-dynamic range (WDR) neurons with both cutaneous and meningeal receptive fields were recognised based on their responses to cutaneous mechanical non-noxious (brushing with a soft brush) and noxious (pinch with forceps) stimulations of their cutaneous receptive fields and to meningeal electrical stimuli delivered through small silver balls.

Recordings were simultaneously made from the ipsilateral LC with a tungsten microelectrode (0.5 MΩ, World Precision Instrument, USA), glued to a glass micropipette for microinjections. Because a rectangular insertion of the recording electrode into the LC would affect the most sensitive part of the meninges and bear a great risk of haemorrhage, the microdrive was tilted to a 30° angle from the horizontal plane, and more anterior coordinates were used (-5.7mm posterior and 0.7mm lateral from bregma). A meningeal incision was made parallel to the transverse sinus at the appropriate coordinates. The electrode was then slowly lowered (6.5–8.2mm) to find the LC. Some elements helped correcting coordinates, namely the mesencephalic trigeminal nucleus neurons, lateral to the LC and responding to jaw opening, as well as tail movements-responsive neurons encountered along the descent (putative cerebellar neurons). LC neurons were identified according to usually described characteristics: tonic activity, biphasic response to noxious stimuli (activation followed by inhibition), and location was later confirmed by a lesion made by passing current through the electrode at the end of the recordings.

LC multi-/single-units, and field potential recordings were both acquired through a Plexon system (on an unfiltered channel for LFP) and the above-described CED 1401plus interface/Spike2 system.

#### Electrical stimulations

Two modalities of electrical stimuli, delivered either to the face (periorbital cutaneous zone) or the meninges, were used: stimuli of 0.8 ms applied every 1.5 s (traditionally used for TCC recordings) or stimuli of 5 ms applied every 2 s (that were previously described as more suitable to see some responses in the LC [24]). The face was stimulated at three times the threshold (in mA) necessary to elicit a C-fibre response in the TCC (0.8 ms stimuli). Given the impossibility to safely map the meningeal receptive field of the TCC WDR because of the near LC electrode, and therefore determine its threshold, intensity of stimulation for meningeal stimuli was kept constant across conditions and animals at 4-5mA.

#### Mechanical stimulations

In all double-site recordings, mechanically evoked LC activity was probed by strong but brief stimulations of the paw and the face. Therefore, we used fine forceps (3 mm apart) to apply mild pressure to the contralateral ankle, or the extremities on the side of the ipsilateral muzzle for a duration of 0.5 s.

For the behavioral/electrophysiological study, we used two types of mechanical stimulations: (i) brushing with a soft brush (0.5-s brush stroke at 0.5 Hz during 20 s) and (ii) pressure applied with VF hairs (0.16, 0.4, 0.6, 1, 1.4, 2, 4, 6 and 8 g), each VF hair being applied once for 5 s in random order, to the most sensitive portion of the cutaneous receptive field.

#### Dural-evoked trigeminal activation

The exposed dura was bathed for 5 min in IS as described before [7].

#### Microinjection (LC)

Propranolol or aCSF microinjections (250 nl) into the LC was carried over 1 min out by applying short pulses of high-pressure air with a custom-made apparatus.

### Patch-clamp electrophysiological recordings

Rats were deeply anaesthetized with an intraperitoneal (i.p.) overdose of chloral hydrate (7%) and decapitated. The brains were quickly removed and immediately chilled in ice-cold cutting-based saline solution bubbled with carbogen (95% O_2_, 5% CO_2_) and containing the following (in mM): 2 KCl, 0.5 CaCl_2_, 7 MgCl_2_, 1.15 NaH_2_PO_4_, 26 NaHCO_3_, 11 glucose, and 205 sucrose. After removal of the dura mater, the brainstem including LC was transversally sliced (350 μm thick) with a vibratome (VT1200 S, Leica Microsystèmes SAS, France). After cutting, slices were incubated at 37 °C in aCSF containing (in mM): 130 NaCl, 3 KCl, 2.5 CaCl_2_, 1.3 MgSO_4_, 0.6 NaH_2_PO_4_, 25 NaHCO_3_, 10 glucose (pH 7.4) bubbled with 95% O_2_ and 5% CO_2_, for a 45 min recovery period. Slices were then transferred into the recording chamber.

LC neurons were visualized using an upright microscope fitted with fluorescence optics (AxioExaminer, Carl Zeiss, Germany) and linked to a digital camera QImaging Exi Aqua (Czech Republic). Patch pipettes (5–7 MΩ resistance), made from borosilicate glass (1.5 mm O.D; PG150T-15; Harvard Apparatus, UK) were filled with an internal solution containing (in mM): 135 KCl, 0.5 mM CaCl_2_, 2 MgCl_2_, 5 KCl, 5 EGTA 5 Hepes, 5 ATP-Na_2_, 0.5 GTP-Na_2_, neurobiotin (0.05%, Vector Laboratories, Burlingame, CA, USA), dextran tetramethylrhodamine (10,000 MW, fluoro-ruby, 0.01%, Life technologies, Saint Aubin, France), pH adjusted to 7.4 and osmolarity of 290–300 mOsm.

Whole-cell voltage-clamp recordings were made at room temperature (22—24°C). Acquisitions were performed using Clampex 10 software (Molecular Devices, Sunnyvale, CA, USA) connected to a Multiclamp 700B amplifier (Molecular Devices, Sunnyvale, CA, USA) via a Digidata 1440A digitizer (Molecular Devices, Sunnyvale, CA, USA). Voltage-clamp data were low pass filtered at 2 kHz and digitized at 10 kHz. Series resistance was monitored at the beginning and end of each recording session, and data were rejected if values changed by > 20%.

At the end of the recordings, epifluorescence was used to ensure that the recorded cells were filled with dextran tetramethylrhodamine. Immunolabeling neurobiotin was carried out to check that the recorded neurons were in LC region. Slices were transferred into 4% paraformaldehyde in 0.1M phosphate-buffered solution (pH 7.4) and stored overnight at 4°C. Next, fixed slices were washed with 0.05 M Tris-buffered saline (TBS), and were incubated with Avidin DCS-rhodamine (1:200, Ref. A-2012; Vector Laboratories, Burlingame, CA, USA) for 4 h at room temperature. Subsequently, all slices were mounted on gelatinized slides in a DPX mounting medium, cover-slipped and conserved at 4°C.

### RNAScope fluorescent in situ hybridization

RNAscope kit was purchased from Bio-Techne. Rats were anesthetized, perfused transcardially with 100–200 mL of PBS followed by 500 mL of 4% RNase free paraformaldehyde. The brains were dissected and post-fixed for 24h following by cryoprotection in sucrose (gradient concentration of 10%, 20%, 30%) at 4˚C. The brains were embedded in Tissue-tek and stored at -80°C, until further processing. Frozen tissue was cut on a cryostat at 15 μm, collected on slides, and processed per the manufacturer’s protocol. Every 5th section of LC was analyzed using Zeiss AxioImager M2 microscope and the software Zen 2.3 lite (Zeiss). The probes used were targeted against the rat genes for *adra2a*, *adrb1* and *adrb2*.

### Drugs and substances

IS consisted of 2 mM of histamine, serotonin, bradykinin, and 0.2 mM of PGE2. Inflammatory mediators were diluted in 10 mM Hepes buffer at pH 5.0 (Sigma-Aldrich) [7, 8]. Propranolol (Sigma-Aldrich) was diluted in aCSF to a concentration of 1 mg.mL^−1^. Vecuronium bromide (Sigma-Aldrich) was diluted in physiological serum (9 g NaCl.mL^−1^ H_2_O), filtered, and stored in 10 mL tubes at -20°C.

### Analysis

Spikes extraction was performed using the built-in Spike2 tools for single- and/or multi-units, and all data were exported from Spike2 to MATLAB (R2014b) for further analysis. Unless otherwise stated, common electrophysiological analyses were performed using custom-made scripts and built-in MATLAB functions; we also used some functions from the FMA-Toolbox (http://fmatoolbox.sourceforge.net/).

#### Single-unit versus multi-unit activity

While for the TCC, we analysed single-unit (SU) activity, results presented for the LC were extracted from multi-unit responses (MUA) unless otherwise stated, for several reasons: (i) single-unit activity (SUA) responses are usually quite low (that is, a cell rarely fires more than 1 or 2 spikes per electrical stimulus), requiring repetitions, but MUA yields the same results with less “noise” (thanks to the uniformity of response of a local population of cells); (ii) the modality of microinjection, first designed for behavioral studies, is hardly compatible with continuous SU recording. From a technical point of view, we extracted as ‘MUA’ cells that were not reliably identifiable as SU but with clearly-defined action potentials, and only kept as occasional ‘SU’ cells that were readily separable according to their amplitude.

#### Electrically-evoked activity within LC and TCC

We adapted a previously described method to extract LC electrically-evoked activity [7]: post-stimulus time histogram (PSTH) were constructed using spikes evoked from desired stimulations, and bins of evoked activity were defined as bins with a frequency of discharge greater than the mean frequency of bins in the last 500 ms of the PSTH. The different components of the response were then identified and quantified as mean frequency of discharge above this last 500 ms epoch, and normalized by baseline frequency (i.e. divided by the mean frequency before the electrical train). A and C responses within TCC were extracted under 30 ms post-stimulus for an A response, and between 30–200 ms post-stimulus for a C response (PSTH were used to confirm in each case that these latencies were appropriate).

#### Mean frequency analysis (spontaneous activity)

Mean frequency was computed using a centred window of defined width (depending on the analysis) at each spike event, and oversampled/decimated to a fix sampling rate. Some results are presented as a total activity evoked by a stimulation: for the TCC, it corresponds to the raw sum of the number of spikes during the appropriate time window, for the LC, it is the raw count of MUA divided by pre-stimulus count so that the result is a percentage of baseline activity.

#### Phase analysis

Mean MUA frequency was first bandpassed (FIR filter), and Hilbert transform was performed to extract the corresponding phase. Phase histograms were then constructed by allocating each spike to the corresponding bin of the phase during which it occurred (75 or 150 bins total for a period). Histograms were z-scored to keep only temporal dynamics and process mean over similar conditions.

#### Power in MUA

The ratio of power in the delta band (0.3 to 2 Hz) to total power of MUA was extracted on a mean frequency of MUA. To compare propranolol effect as before/after, a mean ratio was computed on 10 min between trains of electrical stimulations (two inter-train windows) either before dural injection and microinjection or after microinjection.

#### Local field Potential (LFP)

Raw LFP signals were decimated (low pass filtered and down sampled) down to a sampling frequency of 50 Hz. Spectrograms of decimated signals were generated using Short Time Fourier Transform with the following parameters: Hamming window of length 1024, overlap of 1014 and FFT length of 8096. The log of resulting power spectral density (PSD) was then integrated from 0.3 Hz to 2 Hz to produce a time-varying value reflecting the low frequency activity within the signal. Correlations between this resulting power and the slow variations of MUA mean frequency (slow oscillations) were computed on 400 s sliding windows after smoothing (Savitsky-Golay, order 3, 51 samples), and R^2^ max was extracted for each rat.

Cross-correlograms were used to determine the mean lag between Sp5C and LC LFP signals, the peak giving the value and the direction of the lag.

### Statistics

Comparisons among groups for the behavioral studies were performed by 2-way analysis of variance (ANOVA). When only two sets were compared, normality was checked using Lilliefors test. If the hypothesis of normality was true, Student’s t test (paired or unpaired, according to conditions), otherwise Wilcoxon signed-rank test or Mann–Whitney U test were used.

### Code availability

Computer code used to analyze the datasets is available from the corresponding authors on reasonable request.

### Data availability

The datasets analysed during the current study are available from the corresponding authors on reasonable request.

## Results

### Dural-evoked trigeminal activations lead to phase shifting between LC and TCC spontaneous oscillatory activities

We first investigated the temporal relationship between LC and TCC neuronal spontaneous activities, by simultaneously recording single- and multi-unit activities (SUA and MUA) as well as local field potentials (LFP) in naive rats.

**Fig. 2:**
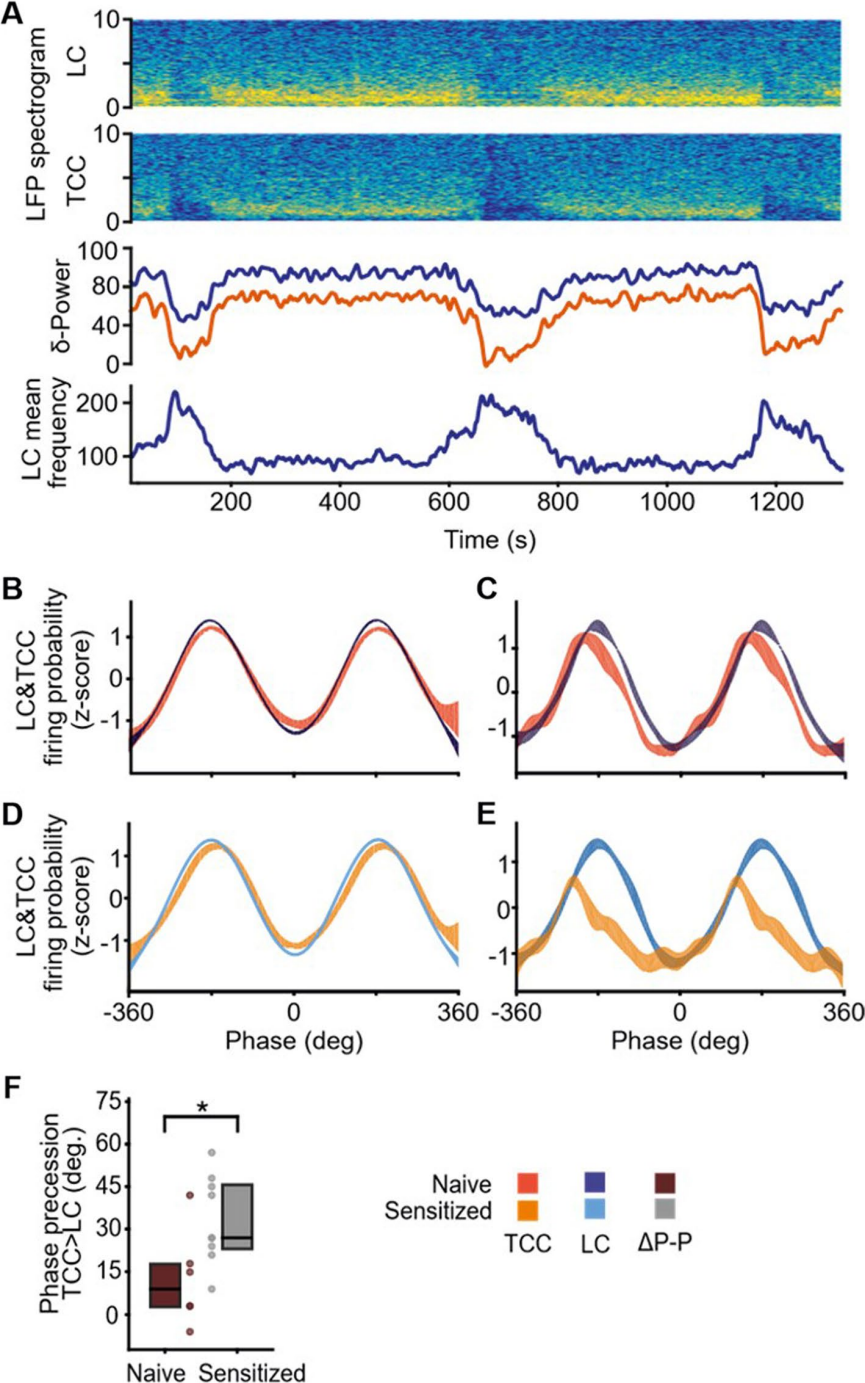
Alterations of trigemino-coerulean spontaneous oscillatory in conditions of dural nociceptor sensitization. **A** Representative examples of spectrograms from LC and TCC LFP recordings (top) and quantification of their delta power (bottom) shown above LC spontaneous activity, presenting slow oscillations. **B** to **E** Z-score of spikes phase preferences (mean ± SEM) calculated to obtain a pure time representation of probability of neuronal discharge, allowing to average across animals. Spikes phase preference (mean ± SEM) of TCC (red) and LC (blue) for intermediate (**B**, *n* = 12) and slow (**C**, *n* = 6) oscillations in naive rats. Spikes phase preference (mean ± SEM) of TCC (yellow) and LC (blue) for intermediate (**D**) and slow (**E**) oscillations in sensitized rats. **F** Precession of TCC preferred phase (slow oscillations) on LC preferred phase in naive (*n* = 6) versus sensitized animals after IS4 (*n* = 9)

As previously shown [25], the MUA of LC spontaneously oscillated, alternating periods of enhanced and reduced activity (Fig. 2A). Temporal analysis of the spontaneous activity revealed different co-existing oscillatory rhythms (Supplementary Fig. 1): slow (period: 400.4 ± 74.6 s), intermediate (period: 21.3 ± 0.5 s) and a third faster one, in the delta range, visible on mean frequency curves. This latter rhythm appeared to vanish when LC spontaneous activity reached its maximum values. TCC neurons usually, but not consistently, exhibited low spontaneous activity as expected from naive animal [7]. Notably, such spontaneous activity followed the very same oscillatory pattern as that of the LC. In particular, the slow and intermediate oscillations of spontaneous TCC and LC neuronal activities were in phase (Fig. 2B and C), suggesting that the spontaneous activities of LC and TCC neurons are synchronized in naive rats.

LFP recordings confirmed this hypothesis since LC as well as TCC neurons were synchronized within the delta band (0.3–2 Hz; Fig. 2A), and exhibited a positive correlation (*r*^*2*^ = 0.74 ± 0.05). Further analysis revealed that, according to LFP activity, the LC preceded the TCC (cross-correlation peak: 10.1 ± 0.6 ms), suggesting that LC spontaneous activity drives that of the TCC.

To characterize LC-TCC relationship in sensitized conditions (after the 4^th^ IS infusion), we analyzed their spontaneous activities after repeated dural IS infusions. Under these conditions, TCC spontaneous activity became enhanced, thus reflecting neuronal sensitization (Supplementary Fig. 2A and [7]). In contrast, LC spontaneous activity remained unchanged (Supplementary Fig. 2B). Nevertheless, TCC and LC spontaneous activities displayed the very same slow and intermediate oscillatory patterns in sensitized as in naive rats (Fig. 2C and E; periods: 675.0 ± 100.7 s and 20.5 ± 0.3 s (sensitized rats); 400.4 ± 74.6 s and 21.3 ± 0.5 s (naïve rats)), consistent with the LC still driving TCC spontaneous neuronal activity. However, the relationship between TCC and LC slow oscillations had changed. Notably, TCC spontaneous activity no more closely paralleled that of LC along its whole oscillatory cycle, as in naive rats, but rather diverged from it when it had reached a maximum value (Fig. 2E). This resulted in a phase shift between the peaks of TCC and LC discharge probabilities, that of the TCC now occurring sooner than that of the LC in sensitized animals (Fig. 2F). Thus, the LC lost partly its ability to drive TCC spontaneous activity when the latter is too high.

### Dural-evoked trigeminal activations lead to TCC sensitization but less LC responsiveness

**Fig. 3:**
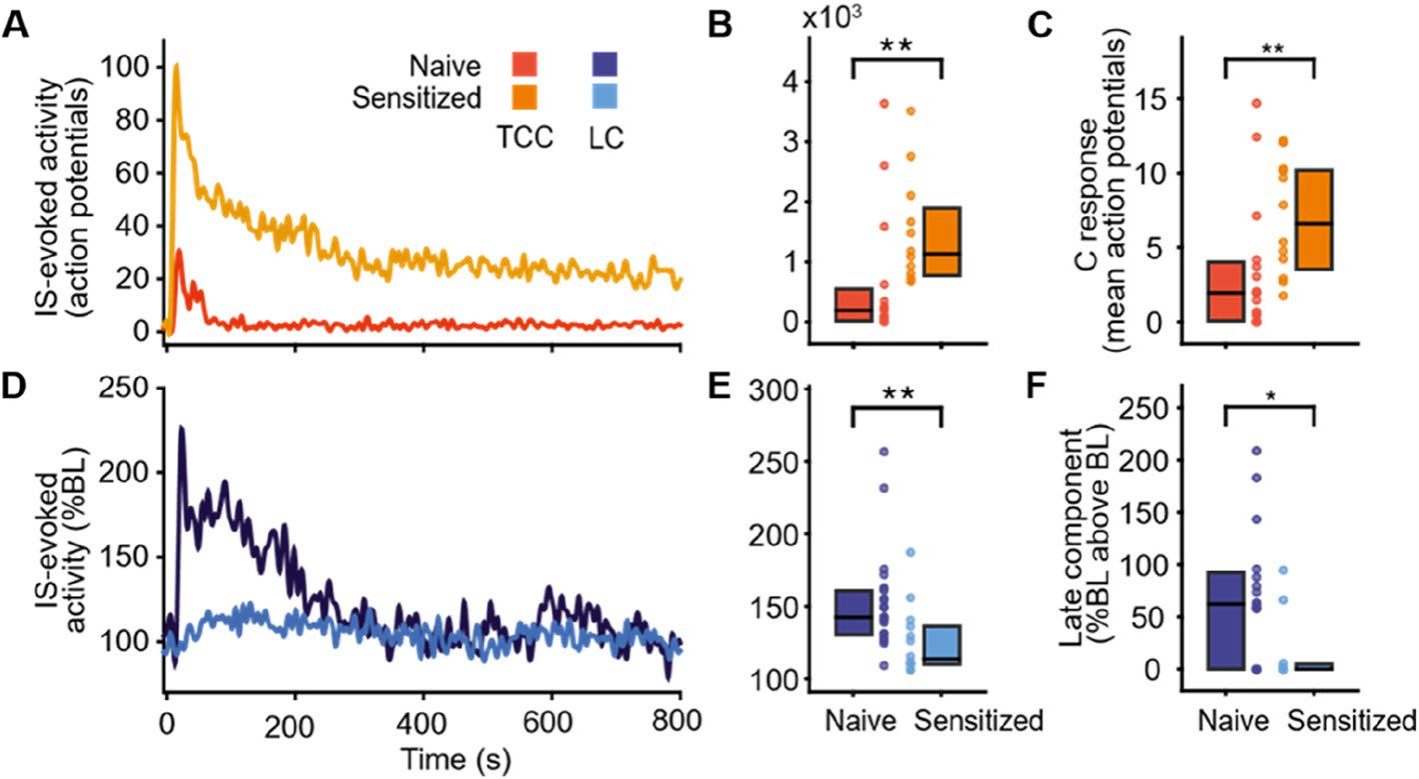
Uncoupled integration of nociceptive information within trigemino-coerulean network in sensitized (IS4) conditions. Individual example (**A**) and cumulative results (**B**) of the activity evoked in TCC by application of IS on the meninges (at t = 0s) of naive (red, *n* = 15) and sensitized (orange, *n* = 12) animals. **C** Mean number of spikes of the C response of TCC evoked by 50 successives electrical stimulations of the meninges in naive (*n* = 15) and sensitized (*n* = 12) animals. Individual example (**D**) and cumulative results (**E**) of the activity evoked in LC by application of IS on the meninges (at t = 0s) of naive (dark blue, *n* = 22) and sensitized (light blue, *n* = 14) animals. **F** Late component within LC evoked by 50 successives electrical stimulations of the meninges in naive (*n* = 16) and sensitized (*n* = 11) animals. Horizontal line in boxplots represents median, bottom and top edges 25th and 75th percentiles respectively, and dots represent individual values. * *P* < 0.05, ** *P* < 0.01,  Mann–Whitney U test

Because LC neurons respond to sensory stimuli of different modalities with phasic activity, which has been suggested to modulate sensory processing [26], we investigated the effects of cephalic cutaneous and meningeal stimulation on TCC and LC neuronal activities. In naive rats, application of electrical suprathreshold stimuli to cutaneous and meningeal receptive fields elicited two separated responses (times to peak: < 30 ms and between 30 and 100 ms) in all recorded TCC neurons (Supplementary Fig. 3A and B), consistent with Aδ- and C-fiber-evoked responses. Such peripheral stimulations also produced an early and late responses in 42% of LC neurons (*n* = 8/19) (Supplementary Fig. 3C and D). The remaining LC neurons (*n* = 11/19) exhibited only the early response. Of note, early and late LC responses to dural stimulation (times to peak: 62.8 ± 3.9 ms, and 129.6 ± 5.3 ms, respectively) were markedly delayed, compared to TCC ones. This delay in LC activity could reflect an integration processing of trigeminal nociceptive information along the TCC—LC pathway. In line with the occurrence of peripheral stimulation-evoked responses in the TCC as well as the LC, an acute dural IS infusion markedly increased action potential frequency in both areas (Fig. 3A, B and D, E).

As previously shown [7], repeated dural IS infusions led to TCC neuron sensitization, manifested as potentiated responses and reduced electrical thresholds respectively to inflammatory (Fig. 3A and B) and electrical stimulation of the meninges (Fig. 3C). On the other hand, LC neurons exhibited reduced, if not suppressed, IS application-induced neuronal activity (Fig. 3D and E) as well as dural electrical stimulation-evoked late responses (Fig. 3F). Thus, repeated dural IS infusions, while sensitizing TCC neurons, appears to conversely attenuate LC responsiveness to meningeal stimulation. Together with our evidence that LC spontaneous activity remains unchanged following repeated dural IS infusions while TCC one increased (Supplementary Fig. 2A and B), this suggests a decoupling of LC and TCC networks under sensitized conditions through a disengagement of LC to meningeal stimulations (Supplementary Fig. 2C).

### Propranolol treatment into the LC prevented TCC sensitization

We previously demonstrated that systemic administration of the beta-blocker propranolol both prevents repeated IS-induced TCC sensitization and decreases stimulus-evoked neuronal activation within the LC [12]. Here, we microinjected propranolol directly into the LC before each IS infusion on the meninges.

**Fig. 4:**
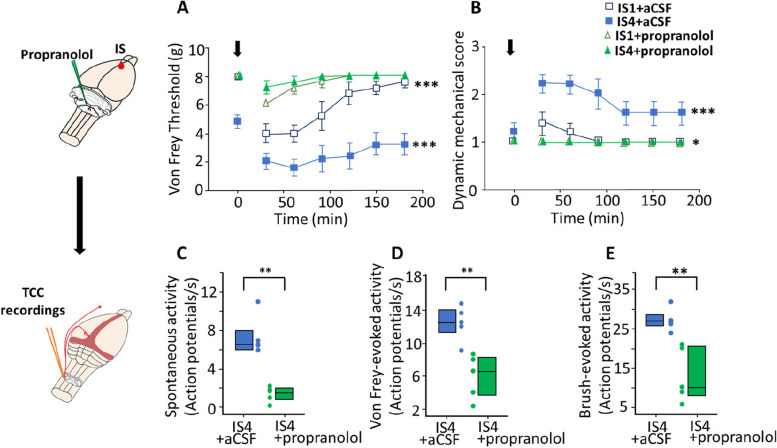
Propranolol microinjections in the LC prevents TCC sensitization and cutaneous allodynia. Left panels: techniques and treatments depicted in each line. **A** Time courses of static mechanical withdrawal thresholds and (**B**) dynamic mechanical scores (DMS) of the face after a 1st (unfilled symbols) or 4th IS (colored symbols) injection in vehicle (squares)- or propranolol-treated rats (triangles). Cutaneous mechanical sensitivity was assessed before and at 30 min intervals for 3 h after IS injection (black arrows at t0). Static mechanical thresholds were measured with VF hairs applied on the face and dynamic mechanical scores by gentle air puffing. (**C**) to (**E**) Electrophysiological recordings (TCC) from the same animals as in (**A**) and (**B**). **C** Spontaneous, (**D**) VF-evoked, and (**E**) brush-evoked activities from IS4 + aCSF (left boxplots) and IS4 + propranolol rats (right boxplots). Horizontal line in boxplots represents median, bottom and top edges 25th and 75th percentiles respectively, and dots represent individual values. For all conditions, *n* = 5. * *P* < 0.05, ** *P* < 0.01, *** *P* < 0.001; two-way ANOVA for (**A**) and (**B**) (aCSF vs propranolol microinjection, on 1st or 4th IS injection), Mann–Whitney U test for (**C**), Student’s t test for (**D**) and (**E**). IS1 = 1st IS injection (naive animals), IS4 = 4th IS injection (sensitized animals) and the corresponding experimental groups

Repeated IS-induced TCC sensitization can be behaviorally assessed by testing cutaneous sensitivity. As previously shown [7, 12], repeated dural IS infusions produced a gradual worsening of inflammation-induced cephalic cutaneous mechanical hypersensitivity (measured right after the 1^st^ and 4^th^ IS infusions or ictal) superimposed on a persistent cutaneous hypersensitivity (measured before the 4^th^ IS infusion or inter-ictal). Such persistent static mechanical allodynia (measured using VF stimuli) appeared after the 3^rd^ IS infusion (Fig. 4A). Dynamic mechanical allodynia (measured using air-puff stimuli) also progressively worsened with IS repetition but never became persistent (Fig. 4B). The 1^st^ propranolol microinjection into the LC prevented the development of both static, at least in part (Fig. 4A), and dynamic (Fig. 4B) cutaneous mechanical hypersensitivities. Notably, the effectiveness of the preventive treatment improved with repeated propranolol microinjections (Fig. 4A, B). Consistent with behavioral results, propranolol treatment also reduced the magnitude of the spontaneous activity (Fig. 4C), as well as electrophysiological responses of TCC neurons to innocuous mechanical stimulations (Fig. 4D, E), compared with those in TCC neurons recorded in aCSF-microinjected animals. Thus, propranolol directly injected into the LC can prevent trigeminal sensitization.

### Propranolol decreased LC neurons excitability, likely through beta-2 adrenergic receptors

**Fig. 5:**
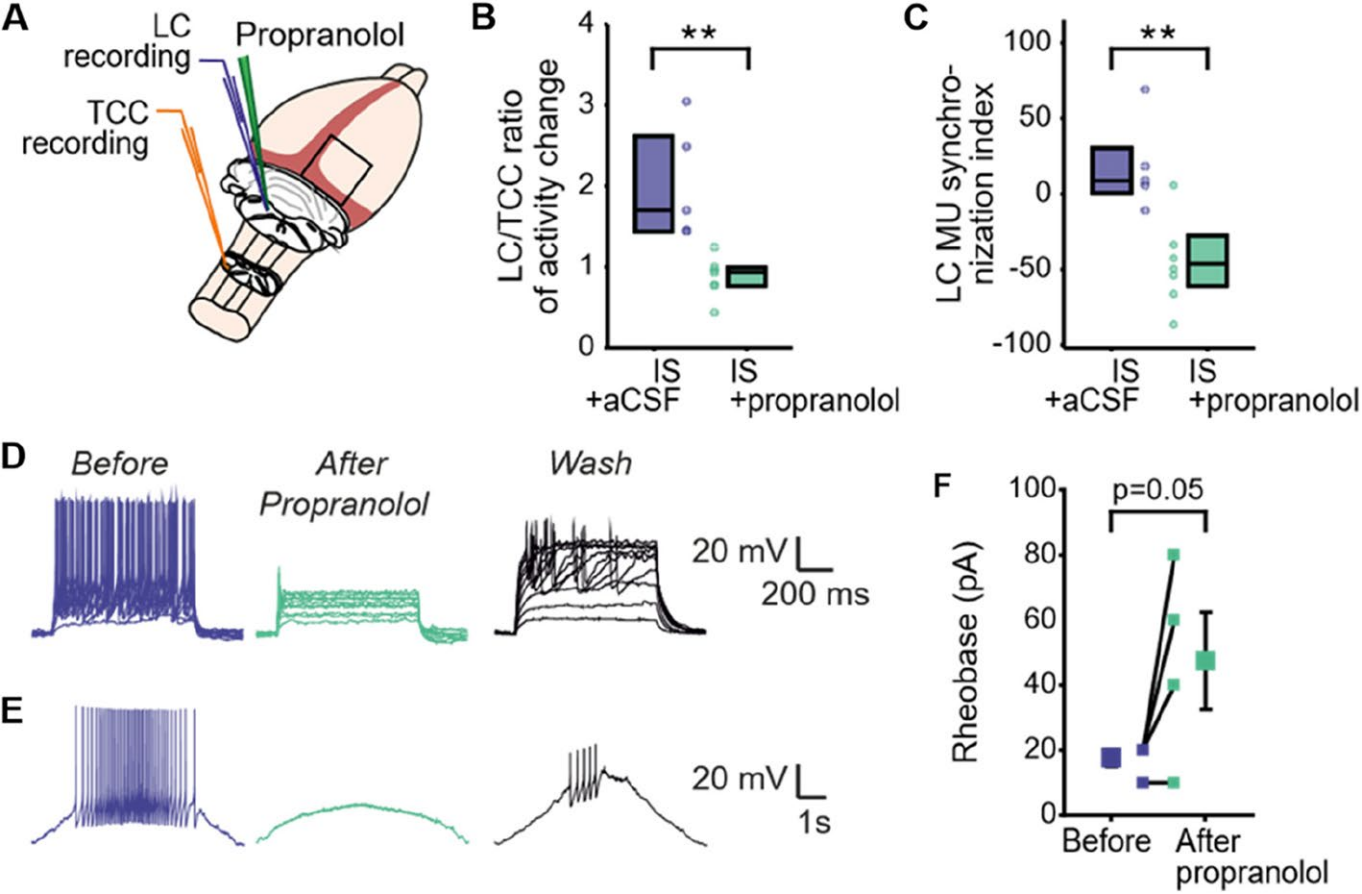
Propranolol dampens LC functioning. **A** Simultaneous recordings from the TCC and the LC were made from naïve rats; after baseline measures, they received either IS (meninges) + aCSF (LC) or IS (meninges) + propranolol (LC), and measures were repeated after 30min. **B** Ratio of LC early component (in percentage of response above BL normalized by BL) to TCC A response (total number of spikes for the train) evoked by cutaneous electrical stimulation after IS and then propranolol or aCSF microinjection in the same animals (*n* = 7). **C** Index of synchronisation among spontaneous LC MUA activity (normalized power in delta frequency) before and after IS and propranolol or aCSF microinjection (*n* = 8). **D**-**E** Examples of firing patterns (current-clamp mode) evoked by depolarizing (**D**) or ramp currents (**E**) before, during propranolol application and after washout. **F** Variation in rheobase before and after bath-applied propranolol (*n* = 4). Symbols in the middle of graphs show values before and after propranolol for each individually recorded neuron. Horizontal line in boxplots represents median, bottom and top edges 25th and 75th percentiles respectively, and dots represent individual values. * *P* < 0.05, ** *P* < 0.01; paired Student’s t test for (**B**) and (**C**)

The robust effects of propranolol microinjections into the LC on TCC sensitization led us to investigate the underlying mechanisms. To address this issue, we assessed the electrophysiological changes in the LC and TCC produced by a single propranolol microinjection into the LC after one IS application onto the meninges of naive rats (Fig. 5A). One microinjection of propranolol significantly decreased the ratio of LC to TCC neuronal activities evoked by cutaneous stimulation (Fig. 5B), that is, the same TCC output produced less activation of LC neurons. In the same time, LC MUA synchronization was also decreased (Fig. 5C).

To assess whether the propranolol has a direct and inhibitory effect on LC neurons, we have recorded LC neurons using ex vivo whole-cell patch-clamp recordings in brainstem slices (Fig. 5D to F). In 75% of LC neurons, bath-applied propranolol reduced the number of action potentials (AP) triggered by depolarizing pulses, transforming regular spiking into single spiking neurons (Fig. 5D, *after propranolol*) and prevented the appearance of APs evoked by depolarizing ramp currents at 2 × the rheobase (Fig. 5E, *after propranolol*). Propranolol application also increased the rheobase (current necessary to obtain action potential in current clamp mode; Fig. 5F). In one neuron, a 30 min-long washout could reverse propranolol effects on LC neurons (Fig. 5D and E, *Wash*). Thus, propranolol application directly decreases the excitability of LC neurons.

**Fig. 6:**
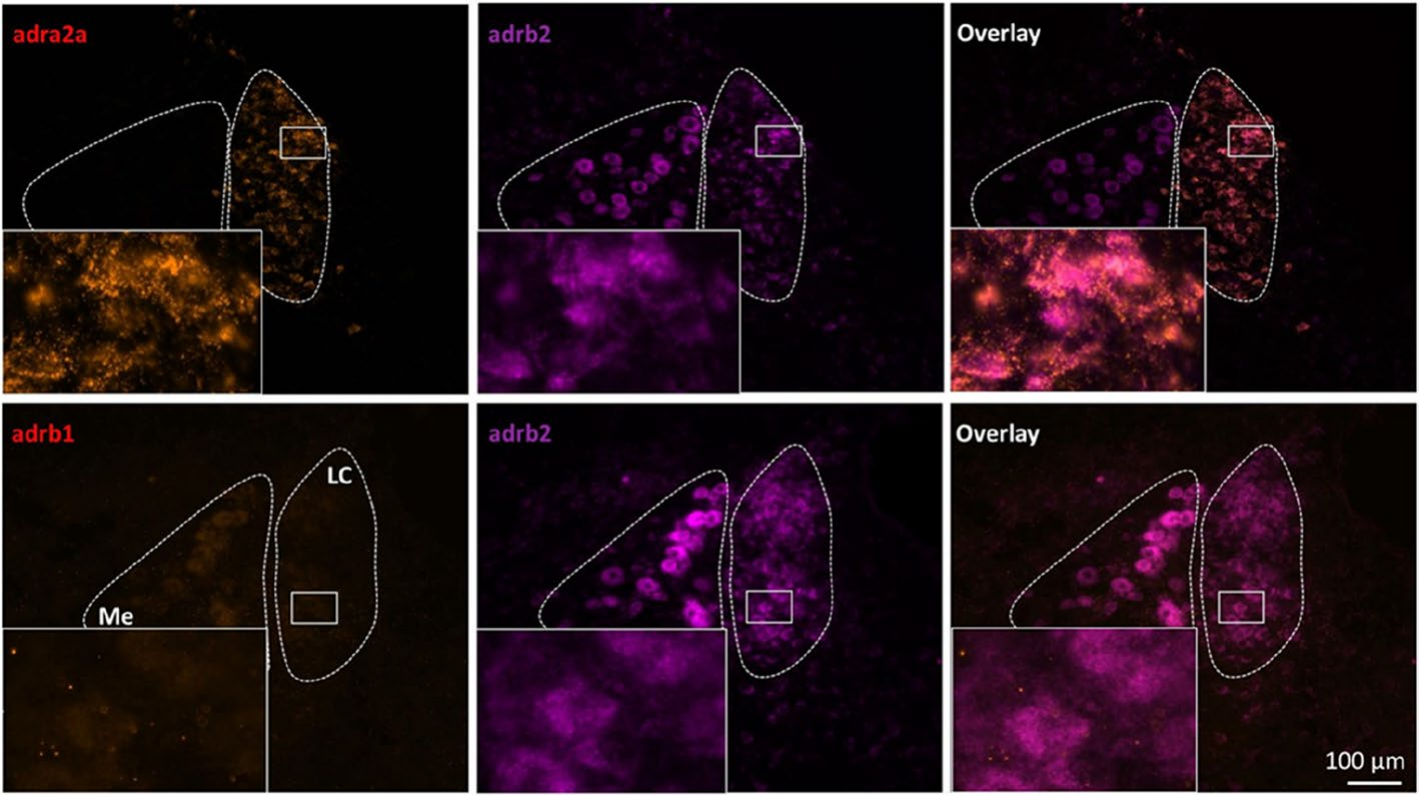
Beta-2 and alpha-2A AR, but not beta-1 AR are co-expressed into LC. In situ hybridization was performed on brain slices from adult rats (*n* = 3) using probes for adra2a (alpha-2A AR, red) and adrb2 (beta-2 AR, magenta) in one set of slices, and adrb1 (beta-1 AR, red) and adrb2 (beta-2 AR, magenta) in a second set of slices. Me: Mesencephalic nucleus; LC: Locus Coeruleus

To explore the potential receptors responsible for the effects of propranolol on LC neurons, we examined the expression of beta-1 and beta-2 AR using RNAscope in situ hybridization (Fig. 6).

As control, a third probe targeting *adra2a* (alpha-2A AR) was used, as this latter receptor is known to be an auto-receptor of LC noradrenergic neurons [21]. As expected, we observed many alpha-2A AR mRNA-positive cells in LC. Because the RNAscope Fluorescent Multiplex assay allows the detection of two targets at the same time, we co-incubated *adrb2* and *adra2a* probes, as well as *adrb1* and *adrb2*, and observed a number of cells co-expressing *adrb2* and *adra2a*. Interestingly, no labelling to *adrb1* was found in LC cells.

Taken together, these findings demonstrate that propranolol decreases the responsiveness of LC neurons to afferent excitatory stimuli and consequently decreases the integration of TCC nociceptive information by LC neurons, likely through beta-2 AR.

## Discussion

This study shows that the functional LC-TCC coupling, involving both LC tonic and phasic activities, is altered by repeated meningeal inflammation. Specifically, repeated dural inflammations decrease the phasic response of LC neurons, but increase the noxious-evoked activity of TCC neurons, leading to central sensitization. Furthermore, we show that dampening LC neuronal activity with propranolol, most likely by acting on beta-2 AR, prevents sensitization of TCC neurons to meningeal and cutaneous stimulations.

### Dural IS infusions as migraine model

Many animal models of migraine, such as the administration of glyceryl trinitrate or isosorbide dinitrate, the triggering of cortical spreading depression, or the application of various substances or peptides on the meninges to induce neurogenic inflammation, have been developed and are currently used to study the pathophysiology of migraine [27–29]. Neurogenic inflammation, characterized by an acute sterile inflammation, would involve processes like vasodilation, mast cell degranulation, platelet aggregation, and the participation of vasoactive peptides and inflammatory mediators such as CGRP, substance P, neurokinin A, along with prostaglandins [30]. However, besides CGRP, the role of other neuropeptides, mast cell degranulation, and plasma extravasation in migraine pathophysiology remains uncertain and needs further research [31]. Until now, evidence of inflammation during individual migraine episodes was limited [27], but PET/MRI brain scan studies have provided supporting evidence for the presence of parenchymal as well as meningeal inflammation in migraine patients [32, 33]. Additionally, supporting a role for neurogenic inflammation in migraineurs, elevated levels of plasmatic IL-1β, prostaglandin E_2_, tumor necrosis factor-α (TNF-α), IL-6, or nitrite were observed during migraine attacks [34–38]. Therefore, even though the dural neurogenenic inflammation model using repeated IS infusions could be more akin to meningitis than migraine itself, it allows us to study the consequences of applying inflammatory substances to the meninges in migraine context. Indeed, it has been demonstrated that the infusion of IS provokes an increase of face rubbing [39] and grooming [40] which are indicative of head pain and/or irritancy in rats. Furthermore, IS infusions increase rest and freezing behavior, and decrease exploratory behavior [41], which are behaviors also experienced by migraine patients. Consequently, this model enables us to investigate the impact of repeated stimulation of meningeal nociceptors, as it occurs during the headache phases in migraine, on peripheral and central sensitization.

### The LC-TCC dialog under physiological conditions

The LC plays key roles in sensory signal processing and notably modulates sensory-nociceptive integration, through both the bottom-up stream that directly conveys sensory information and the top-down control signals [9, 42–44]. While studies showed that LC phasic activity is involved in pain modulation [45–48], none of them has investigated both tonic and phasic activities of LC, using multi-site electrophysiological recordings. Here we show that LC and TCC spontaneous activities are correlated. LC spontaneous spiking, i.e. tonic activity, and LFPs exhibited spontaneous oscillations in naive conditions, likely due to synchronization of LC neurons via gap-junctions [49]. A model of the resulting electrotonic coupling predicts that such synchrony should disappear at higher discharge rates [50]. Consistently, LFP recordings exhibited a delta synchrony, which decreased when LC spontaneous activity reached maximum values. That LC LFPs precede TCC ones and that LC oscillations are present even without TCC spontaneous activity suggest that LC drives TCC neuronal excitability.

In parallel to this descending modulation of TCC spontaneous activity, LC also integrates ascending trigeminal sensory information. In naive conditions, meningeal as well as cutaneous cephalic stimulations reliably evoked LC phasic discharges. However, for electrical stimulations that consistently generated A and C fiber-evoked responses in TCC, a late response in the LC was less robustly observed. As LC cellular composition is heterogenous [51, 52], and can be segregated with respect to efferent targets function [53], we may have recorded different types of neurons within the LC, with variable sensitivity to cephalic peripheral inputs. The chemical meningeal stimulation activated also TCC as well as LC neurons. We and others have previously shown that this stimulation leads to TCC sensitization, manifested as behavioral mechanical allodynia [7, 8, 54]. Here we addressed the role of the LC in such sensitization, knowing that the LC is involved in facilitating as well as inhibiting pain. Thus, increasing LC phasic activity by electrical [45–47], chemogenetic [48] or optogenetic [44, 52] stimulations leads to an analgesic effect. On the other hand, intrathecal noradrenaline led to pain hypersensitivity through activation of specific astrocytes [55]. Likewise, an acute lesion of the LC inhibits meningeal evoked TCC activation [56], as well as the selective destruction of noradrenergic neurons reduces formalin-induced nociception [57–59]. Our results are in line with these latter data since reducing LC activity by propranolol micro-injection dampened the development of cutaneous mechanical allodynia. This suggests that the noradrenergic facilitating pathway prevails over the inhibiting one in acute conditions. Hence, as in other sensory systems, heightened noradrenergic transmission might amplify responses of meningeal nociceptive neurons, to facilitate the transfer of salient or task-relevant stimuli, such as noxious stimuli, to higher order-centers [60].

### The LC-TCC dialog under sensitized conditions

Under sensitized conditions, LC and TCC spontaneous activities were still positively synchronized, but only at the rising phase. Then, TCC one started to decrease while LC one continued to increase, as evidenced by the earlier peak of TCC spontaneous activity compared to LC. Importantly, although it was only very slightly, the same phenomenon was observed for the slow oscillations of LC and TCC spontaneous activities under naive conditions. Our hypothesis to account for this biphasic interplay of LC and TCC spontaneous activities is that TCC spontaneous activity can only reach a maximum due to intrinsic properties of TCC neuronal circuits, and that such maximum occurs sooner in sensitized than in naive conditions as TCC spontaneous activity is then already potentiated.

Concerning the ascending trigeminal sensory information, after repeated meningeal stimulations, responses to additional inflammatory or electrical stimulations of the meninges were increased in the TCC, in agreement with previous reports [7, 8], but decreased strongly in the LC. Therefore, TCC and LC responses to meningeal stimulation became uncoupled, TCC sensitization being associated with LC de-activation. In neuropathic rats too, sensory-evoked responses to noxious stimuli in the LC are significantly altered [61]. This habituation of LC response is not pain specific. Indeed, LC responses to sensory stimulations, such as auditory or visual stimuli, rapidly decrease or even disappear if the stimulus is not followed by reinforcement [62, 63]. It is thus plausible that such adaptation phenomena occur after repeated pain stimulations. But modulation of LC responsiveness to noxious inputs is expected to spill over to the TCC via norepinephrine release. Therefore, lowered LC excitability, following repetitive meningeal stimulations, would actively counteract such enhanced norepinephrine release, and by this fact would decrease the noradrenergic modulation of meningeal nociceptive information in the TCC. The mechanisms of such activity-dependent adaptive changes in the responsiveness of the LC to noxious inputs remain to be determined.

### Propranolol decreases LC responsiveness likely by blocking beta-2 AR

Propranolol is a beta-blocker used as a prophylactic treatment for migraine. In our study, we showed that its direct application into the LC limited the development of cutaneous allodynia induced by repeated meningeal stimulations. This effect appears to be mediated by inhibition of LC activity, since *ex-vivo* electrophysiological recordings showed that propranolol reduces the probability of LC neurons eliciting action potentials. This effect is very similar to what has been obtained with antidepressants, such as desipramine, whose systemic injection causes a decrease in the tonic and phasic activity of LC neurons in both sham and neuropathic animals [61]. Although more work is needed to clarify the underlying mechanism for these effects, desipramine may act by potentiating alpha-2 AR [64], while propranolol is thought to act by blocking beta receptors [19, 20]. For the first time to our knowledge, we show that the auto-receptors modulating noradrenergic neuron activity are not only of alpha-2 AR type. Indeed, our RNAscope results highlighted co-localization of alpha-2A and beta-2 AR mRNAs in LC neurons. So it seems that the tonic and phasic activities of these neurons might be regulated by the balance of these 2 types of receptors. Beta-1 AR mRNA was barely observed in the LC, or its expression is too low to be detected in naive conditions. Alpha-2A AR, associated with a Gi protein, have been implicated in regulating the activity of LC neurons, by inhibiting them [21, 65, 66] and thus inhibiting noradrenaline secretion. Intracellular beta ARs activates, via stimulatory G proteins, adenylate cyclase and increases production of intracellular cAMP [67]. In addition, beta ARs can also activate ERK through a G protein-independent mechanism mediated by β-arrestin [68–70]. However so far, the role of Beta-2 AR is unknown in LC. In amygdala, beta-2 AR activation has been shown to increase neuronal excitability [71], while this receptor enhances long term potentiation in hippocampus [72]. Beta-2 AR have been also involved in pain modulation, acting on different cell types to produce anti-nociceptive [73–76] or pro-nociceptive [77–81] effects. A key aspect of future studies will be to better understand the role of beta-2 AR in the LC.

In summary, our results demonstrate that LC activation, through modulating gain, facilitates the processing of afferent nociceptive information by TCC neurons in normal/physiological conditions, and promotes trigeminal sensitization under acute and repeated meningeal nociceptors stimulations. If we extrapolate this data to the field of migraine, our study demonstrates a direct LC effect on TCC sensitization, in addition to its traditionally recognized role on vasodilatory processes, which also lowers the headache threshold and facilitates induction of migraine attacks [82, 83]. This also adds to its well-established role in gating and modulating sensory information [84], which likely contributes to sensory hypersensitivity. Increased LC responsiveness may thus promote unpleasant sensory symptoms encompassing hallmark migraine symptoms such as photophobia and phonophobia as well as headache attacks in migraine patients. Our study also establishes an LC-dependent mechanism underlying the efficacy of propranolol, which has not been explained to this day. By blocking beta-2 AR, propranolol reduces LC responsiveness to different inputs output and as a result, LC facilitation over TCC. Taken together, our data support the conclusion that effectiveness of propranolol in treatment of migraine is mediated via decreasing LC output, by reducing both its responsiveness and its intrinsic synchronization.

### Supplementary Information

**Additional file 1.**

## Data Availability

The datasets analysed during the current study are available from the corresponding authors on reasonable request.

## Abbreviations

aCSF: Artificial cerebrospinal fluid
AP: Action potentials
AR: Adrenergic receptors
DMS: Dynamic mechanical scores
IS: Inflammatory soup
LC: Locus coeruleus
LFP: Local field potentials
MUA: Multi-unit activity
NO: Nitric oxide
PSTH: Post-stimulus time histogram
SUA: Single unit activity
TCC: Trigeminocervical complex
VF: Von Frey hairs
WDR: Wide-dynamic-range neurons

## References

[1] GBD (2017). Disease and Injury Incidence and Prevalence Collaborators (2018) Global, regional, and national incidence, prevalence, and years lived with disability for 354 diseases and injuries for 195 countries and territories, 1990–2017: a systematic analysis for the global burden of disease study 2017. Lancet.

[2] Ashkenazi A, Sholtzow M, Shaw JW (2007). Identifying cutaneous allodynia in chronic migraine using a practical clinical method. Cephalalgia.

[3] Lipton RB, Bigal ME, Ashina S (2008). Cutaneous allodynia in the migraine population. Ann Neurol.

[4] Schwedt TJ, Krauss MJ, Frey K, Gereau RW (2011). Episodic and chronic migraineurs are hypersensitive to thermal stimuli between migraine attacks. Cephalalgia.

[5] Bigal ME, Lipton RB (2008). Clinical course in migraine: conceptualizing migraine transformation. Neurology.

[6] Louter MA, Bosker JE, van Oosterhout WPJ (2013). Cutaneous allodynia as a predictor of migraine chronification. Brain.

[7] Boyer N, Dallel R, Artola A, Monconduit L (2014). General trigeminospinal central sensitization and impaired descending pain inhibitory controls contribute to migraine progression. Pain.

[8] Burstein R, Yamamura H, Malick A, Strassman AM (1998). Chemical Stimulation of the Intracranial dura induces enhanced responses to facial stimulation in brain stem trigeminal neurons. J Neurophysiol.

[9] Llorca-Torralba M, Borges G, Neto F (2016). Noradrenergic locus coeruleus pathways in pain modulation. Neuroscience.

[10] Pertovaara A (2013). The noradrenergic pain regulation system: a potential target for pain therapy. Eur J Pharmacol.

[11] Howorth PW, Teschemacher AG, Pickering AE (2009). Retrograde adenoviral vector targeting of nociresponsive pontospinal noradrenergic neurons in the rat in vivo. J Comp Neurol.

[12] Boyer N, Signoret-Genest J, Artola A (2017). Propranolol treatment prevents chronic central sensitization induced by repeated dural stimulation. Pain.

[13] Ayata C, Jin H, Kudo C (2006). Suppression of cortical spreading depression in migraine prophylaxis. Ann Neurol.

[14] Kurauchi Y, Haruta M, Tanaka R (2019). Propranolol prevents cerebral blood flow changes and pain-related behaviors in migraine model mice. Biochem Biophys Res Commun.

[15] Sharifpanah F, Saliu F, Bekhite MM (2014). β-Adrenergic receptor antagonists inhibit vasculogenesis of embryonic stem cells by downregulation of nitric oxide generation and interference with VEGF signalling. Cell Tissue Res.

[16] Akerman S, Williamson DJ, Hill RG, Goadsby PJ (2001). The effect of adrenergic compounds on neurogenic dural vasodilatation. Eur J Pharmacol.

[17] Markowitz S, Saito K, Moskowitz MA (1988). Neurogenically mediated plasma extravasation in dura mater: effect of ergot alkaloids. A possible mechanism of action in vascular headache. Cephalalgia.

[18] Reiter MJ (2004). Cardiovascular drug class specificity: beta-blockers. Prog Cardiovasc Dis.

[19] Dahlöf C, Engberg G, Svensson TH (1981). Effects of beta-adrenoceptor antagonists on the firing rate of noradrenergic neurones in the locus coeruleus of the rat. Naunyn Schmiedebergs Arch Pharmacol.

[20] Svensson TH, Almgren O, Dahlöf C (1980). alpha- and beta-adrenoreceptor-mediated control of brain noradrenaline neurons and antihypertensive therapy. Clin Sci (Lond).

[21] Callado LF, Stamford JA (1999). Alpha2A- but not alpha2B/C-adrenoceptors modulate noradrenaline release in rat locus coeruleus: voltammetric data. Eur J Pharmacol.

[22] Zimmermann M (1983). Ethical guidelines for investigations of experimental pain in conscious animals. Pain.

[23] Vos BP, Strassman AM, Maciewicz RJ (1994). Behavioral evidence of trigeminal neuropathic pain following chronic constriction injury to the rat’s infraorbital nerve. J Neurosci.

[24] Hirata H, Aston-Jones G (1994). A novel long-latency response of locus coeruleus neurons to noxious stimuli: mediation by peripheral C-fibers. J Neurophysiol.

[25] Aston-Jones G, Bloom FE (1981). Activity of norepinephrine-containing locus coeruleus neurons in behaving rats anticipates fluctuations in the sleep-waking cycle. J Neurosci.

[26] Devilbiss DM, Waterhouse BD (2011). Phasic and tonic patterns of locus coeruleus output differentially modulate sensory network function in the awake rat. J Neurophysiol.

[27] Reducha PV, Edvinsson L, Haanes KA (2022). Could experimental inflammation provide better understanding of migraines?. Cells.

[28] Harriott AM, Strother LC, Vila-Pueyo M, Holland PR (2019). Animal models of migraine and experimental techniques used to examine trigeminal sensory processing. J Headache Pain.

[29] Tardiolo G, Bramanti P, Mazzon E (2019). Migraine: experimental models and novel therapeutic approaches. Int J Mol Sci.

[30] Matsuda M, Huh Y, Ji R-R (2019). Roles of inflammation, neurogenic inflammation, and neuroinflammation in pain. J Anesth.

[31] Riesco N, Cernuda-Morollón E, Pascual J (2017). Neuropeptides as a marker for chronic headache. Curr Pain Headache Rep.

[32] Albrecht DS, Mainero C, Ichijo E (2019). Imaging of neuroinflammation in migraine with aura: a [11C]PBR28 PET/MRI study. Neurology.

[33] Hadjikhani N, Albrecht DS, Mainero C (2020). Extra-axial inflammatory signal in parameninges in migraine with visual aura. Ann Neurol.

[34] Sarchielli P, Alberti A, Codini M (2000). Nitric oxide metabolites, prostaglandins and trigeminal vasoactive peptides in internal jugular vein blood during spontaneous migraine attacks. Cephalalgia.

[35] Sarchielli P, Alberti A, Vaianella L (2004). Chemokine levels in the jugular venous blood of migraine without aura patients during attacks. Headache.

[36] Sarchielli P, Alberti A, Baldi A (2006). Proinflammatory cytokines, adhesion molecules, and lymphocyte integrin expression in the internal jugular blood of migraine patients without aura assessed ictally. Headache.

[37] Perini F, D’Andrea G, Galloni E (2005). Plasma cytokine levels in migraineurs and controls. Headache.

[38] Yamanaka G, Hayashi K, Morishita N (2023). Experimental and clinical investigation of cytokines in migraine: a narrative review. Int J Mol Sci.

[39] Zhu P, Dong X, Xu H (2021). Microglial P2Y14 receptor contributes to central sensitization following repeated inflammatory dural stimulation. Brain Res Bull.

[40] Hu G, Zhang M, Su M (2018). Wider range of allodynia in a rat model of repeated dural nociception compared with infraorbital nerve chronic constriction injury. Neurosci Lett.

[41] Melo-Carrillo A, Lopez-Avila A (2013). A chronic animal model of migraine, induced by repeated meningeal nociception, characterized by a behavioral and pharmacological approach. Cephalalgia.

[42] Pertovaara A (2006). Noradrenergic pain modulation. Prog Neurobiol.

[43] Alba-Delgado C, Mico JA, Berrocoso E (2021). Neuropathic pain increases spontaneous and noxious-evoked activity of locus coeruleus neurons. Prog Neuropsychopharmacol Biol Psychiatry.

[44] Hirschberg S, Li Y, Randall A (2017). Functional dichotomy in spinal- vs prefrontal-projecting locus coeruleus modules splits descending noradrenergic analgesia from ascending aversion and anxiety in rats. Elife.

[45] Hodge CJ, Apkarian AV, Stevens R (1981). Locus coeruleus modulation of dorsal horn unit responses to cutaneous stimulation. Brain Res.

[46] Jones SL, Gebhart GF (1986). Quantitative characterization of ceruleospinal inhibition of nociceptive transmission in the rat. J Neurophysiol.

[47] Jones SL, Gebhart GF (1986). Characterization of coeruleospinal inhibition of the nociceptive tail-flick reflex in the rat: mediation by spinal alpha 2-adrenoceptors. Brain Res.

[48] Suárez-Pereira I, Llorca-Torralba M, Bravo L (2022). The role of the locus coeruleus in pain and associated stress-related disorders. Biol Psychiatry.

[49] Ishimatsu M, Williams JT (1996). Synchronous activity in locus coeruleus results from dendritic interactions in pericoerulear regions. J Neurosci.

[50] Alvarez VA, Chow CC, Van Bockstaele EJ, Williams JT (2002). Frequency-dependent synchrony in locus ceruleus: role of electrotonic coupling. Proc Natl Acad Sci U S A.

[51] Uematsu A, Tan BZ, Ycu EA (2017). Modular organization of the brainstem noradrenaline system coordinates opposing learning states. Nat Neurosci.

[52] Hickey L, Li Y, Fyson SJ (2014). Optoactivation of locus ceruleus neurons evokes bidirectional changes in thermal nociception in rats. J Neurosci.

[53] Loughlin SE, Foote SL, Bloom FE (1986). Efferent projections of nucleus locus coeruleus: topographic organization of cells of origin demonstrated by three-dimensional reconstruction. Neuroscience.

[54] Yamamura H, Malick A, Chamberlin NL, Burstein R (1999). Cardiovascular and neuronal responses to head stimulation reflect central sensitization and cutaneous allodynia in a rat model of migraine. J Neurophysiol.

[55] Kohro Y, Matsuda T, Yoshihara K (2020). Spinal astrocytes in superficial laminae gate brainstem descending control of mechanosensory hypersensitivity. Nat Neurosci.

[56] Vila-Pueyo M, Strother LC, Kefel M (2019). Divergent influences of the locus coeruleus on migraine pathophysiology. Pain.

[57] Martin WJ, Gupta NK, Loo CM (1999). Differential effects of neurotoxic destruction of descending noradrenergic pathways on acute and persistent nociceptive processing. Pain.

[58] Taylor BK, Roderick RE, Basbaum AI (2000). Brainstem noradrenergic control of nociception is abnormal in the spontaneously hypertensive rat. Neurosci Lett.

[59] Taylor BK, Westlund KN (2017). The noradrenergic locus coeruleus as a chronic pain generator. J Neurosci Res.

[60] Aston-Jones G, Cohen JD (2005). An integrative theory of locus coeruleus-norepinephrine function: adaptive gain and optimal performance. Annu Rev Neurosci.

[61] Alba-Delgado C, Mico JA, Sánchez-Blázquez P, Berrocoso E (2012). Analgesic antidepressants promote the responsiveness of locus coeruleus neurons to noxious stimulation: implications for neuropathic pain. Pain.

[62] Vankov A, Hervé-Minvielle A, Sara SJ (1995). Response to novelty and its rapid habituation in locus coeruleus neurons of the freely exploring rat. Eur J Neurosci.

[63] Hervé-Minvielle A, Sara SJ (1995). Rapid habituation of auditory responses of locus coeruleus cells in anaesthetized and awake rats. NeuroReport.

[64] Cottingham C, Jones A, Wang Q (2012). Desipramine selectively potentiates norepinephrine-elicited ERK1/2 activation through the α2A adrenergic receptor. Biochem Biophys Res Commun.

[65] Wise A, Watson-Koken MA, Rees S (1997). Interactions of the alpha2A-adrenoceptor with multiple Gi-family G-proteins: studies with pertussis toxin-resistant G-protein mutants. Biochem J.

[66] Wang B, Wang Y, Wu Q (2017). Effects of α2A adrenoceptors on norepinephrine secretion from the locus coeruleus during chronic stress-induced depression. Front Neurosci.

[67] Hertz L, Chen Y, Gibbs ME (2004). Astrocytic adrenoceptors: a major drug target in neurological and psychiatric disorders?. Curr Drug Targets CNS Neurol Disord.

[68] Drake MT, Violin JD, Whalen EJ (2008). beta-arrestin-biased agonism at the beta2-adrenergic receptor. J Biol Chem.

[69] Shenoy SK, Drake MT, Nelson CD (2006). beta-arrestin-dependent, G protein-independent ERK1/2 activation by the beta2 adrenergic receptor. J Biol Chem.

[70] Shukla AK, Xiao K, Lefkowitz RJ (2011). Emerging paradigms of β-arrestin-dependent seven transmembrane receptor signaling. Trends Biochem Sci.

[71] Fink AE, LeDoux JE (2018). β-Adrenergic enhancement of neuronal excitability in the lateral amygdala is developmentally gated. J Neurophysiol.

[72] Qian H, Patriarchi T, Price JL (2017). Phosphorylation of Ser1928 mediates the enhanced activity of the L-type Ca2+ channel Cav1.2 by the β2-adrenergic receptor in neurons. Sci Signaling.

[73] Bohren Y, Tessier L-H, Megat S (2013). Antidepressants suppress neuropathic pain by a peripheral β2-adrenoceptor mediated anti-TNFα mechanism. Neurobiol Dis.

[74] Kremer M, Yalcin I, Goumon Y (2018). A dual noradrenergic mechanism for the relief of neuropathic allodynia by the antidepressant drugs duloxetine and amitriptyline. J Neurosci.

[75] Yalcin I, Tessier L-H, Petit-Demoulière N (2010). Chronic treatment with agonists of beta(2)-adrenergic receptors in neuropathic pain. Exp Neurol.

[76] Arora V, Morado-Urbina CE, Gwak YS (2021). Systemic administration of a β2-adrenergic receptor agonist reduces mechanical allodynia and suppresses the immune response to surgery in a rat model of persistent post-incisional hypersensitivity. Mol Pain.

[77] Chen X, Levine JD (2005). Epinephrine-induced excitation and sensitization of rat C-fiber nociceptors. J Pain.

[78] Khasar SG, McCarter G, Levine JD (1999). Epinephrine produces a beta-adrenergic receptor-mediated mechanical hyperalgesia and in vitro sensitization of rat nociceptors. J Neurophysiol.

[79] Ciszek BP, O’Buckley SC, Nackley AG (2016). Persistent catechol-O-methyltransferase-dependent pain is initiated by peripheral β-adrenergic receptors. Anesthesiology.

[80] Li W, Shi X, Wang L (2013). Epidermal adrenergic signaling contributes to inflammation and pain sensitization in a rat model of complex regional pain syndrome. Pain.

[81] Nackley AG, Tan KS, Fecho K (2007). Catechol-O-methyltransferase inhibition increases pain sensitivity through activation of both beta2- and beta3-adrenergic receptors. Pain.

[82] Ashina M (2012). Vascular changes have a primary role in migraine. Cephalalgia.

[83] Goadsby PJ, Lambert GA, Lance JW (1982). Differential effects on the internal and external carotid circulation of the monkey evoked by locus coeruleus stimulation. Brain Res.

[84] Berridge CW, Waterhouse BD (2003). The locus coeruleus–noradrenergic system: modulation of behavioral state and state-dependent cognitive processes. Brain Res Rev.

