# Supplementary material for: Compromised trigemino-coerulean coupling in migraine sensitization can be prevented by blocking beta-receptors in the locus coeruleus
Source: J Headache Pain. 2023 Dec 8;24(1):165. doi: 10.1186/s10194-023-01691-1 (PMC10704784; doi:10.1186/s10194-023-01691-1)
Supplement: Supplementary file 1 — Additional file 1. [file 10194_2023_1691_MOESM1_ESM.docx]

Supplementary Figure 1


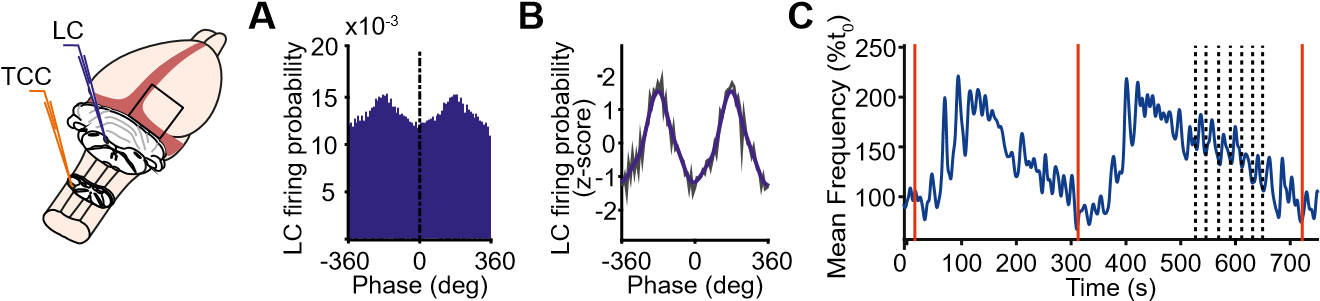


**Supplementary figure 1 – Spontaneous oscillatory activity in LC.** (**A**) The probability of discharge of LC MUA, that can be seen as the global distribution of spikes in (**C**), was calculated in function of intermediate and slow oscillations phases. (**B**) Histogram in (**A**) was z-scored to obtain a pure time representation of probability allowing to average across animals. (**C**) Example of mean spontaneous multi-unit activity (MUA) in LC expressed as percentage of t_0_ activity. Red vertical lines delineate two slow oscillations, dashed lines delimit a subset of intermediate oscillations.

Supplementary Figure 2


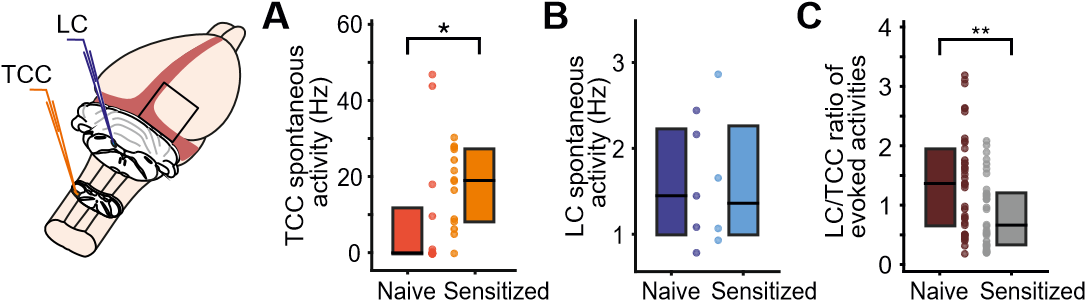


**Supplementary figure 2 – Spontaneous activities in TCC and LC in naive and sensitized rats.** (**A**) Mean spontaneous activity of TCC cells in naïve (n = 13) *versus* sensitized (n = 14) animals. (**B**) Mean spontaneous activity of LC cells in naïve (n = 5) *versus* sensitized (n = 4) animals. (**C**) Ratio of LC early component (in percentage of response above BL normalized by BL) to TCC A response (total number of spikes for the train) evoked by cutaneous electrical stimulation in naïve (*n* = 11) and sensitized (*n* = 11) animals. Horizontal line in boxplots represents median, bottom and top edges 25th and 75th percentiles respectively, and dots represent individual values. * P < 0.05, Mann–Whitney U test for (**A, C**) and Student’s t test for (**B**).

Supplementary Figure 3


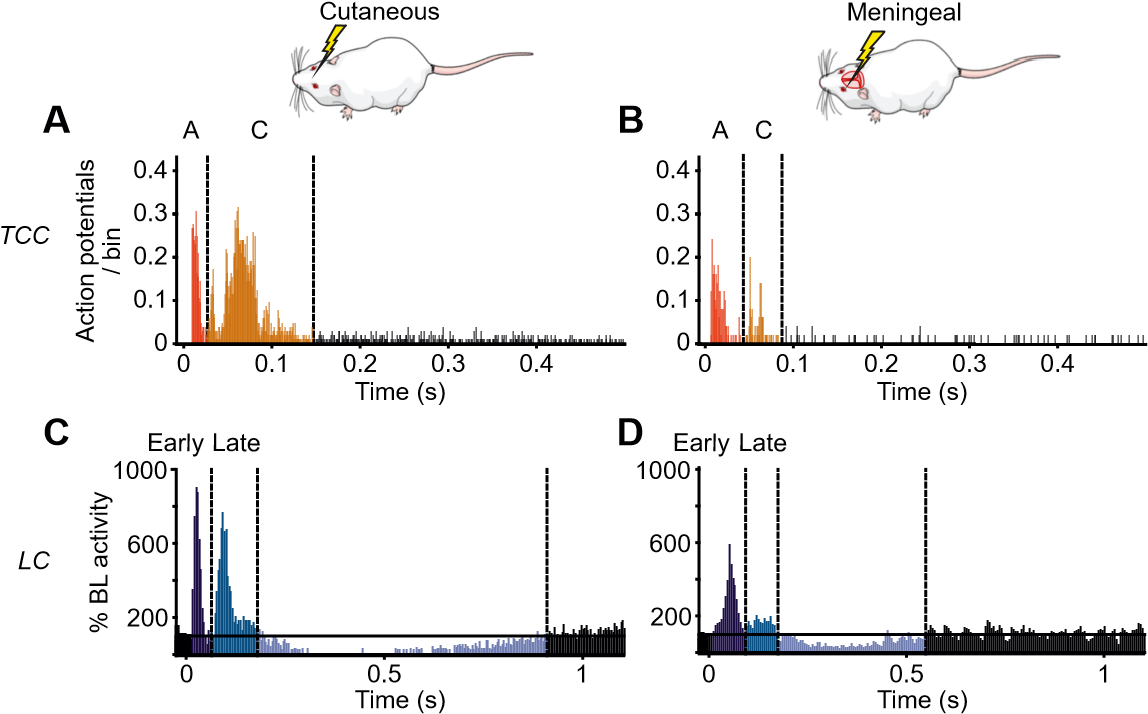


**Supplementary figure 3 – TCC and LC integrate cutaneous and meningeal nociceptive information.** (**A**) to (**D**) Typical examples of PSTH obtained by applying repeated electrical stimuli (*t* = 0 s) either to meninges (near the veinous sinus as shown by the black square in Figure 1B) or cutaneous territory while recording both TCC (**A**, **B**) and LC (**C**, **D**). TCC PSTH, bins of 1ms; LC PSTH, bins of 5ms. Vertical dashed lines show the limits between the different components of the response: A and C responses for TCC, and the corresponding early and late responses for the LC. Note the differences of abscissa scales, adjusted to clearly show all components.
